# Coexistence of *β*-Lactamases in Community-Acquired Infections in a Tertiary Care Hospital in India

**DOI:** 10.1155/2019/7019578

**Published:** 2019-12-11

**Authors:** Shahzad Mirza, Savita Jadhav, R. N. Misra, Nikunja Kumar Das

**Affiliations:** Department of Microbiology, Dr., D. Y. Patil Medical College Hospital and Research Centre, Pimpri, Pune 18, India

## Abstract

**Introduction:**

The trends of *β*-lactamases producing *Enterobacteriaceae* is ever increasing, and limited studies have reported investigating coexistence of *β* lactamases in *Enterobacteriaceae*. A cross-sectional study after approval from the Institutional Ethical committee was conducted between June 2014 and May 2016 in community-acquired infections due to multidrug-resistant organisms in our tertiary care. Nonrepetitive clinical samples from the out-patient department (OPD) were processed for bacteriological culture and identification of *Enterobacteriaceae.* An antibiotic susceptibility test, screening, and phenotypic confirmation for ESBLs and carbapenemases and AmpC producers were performed to check for coexistence of these enzymes.

**Results:**

Nonrepetitive clinical specimens processed for culture and identification in our hospital revealed 417 positive isolates in community acquired infections which were multidrug-resistant organisms, and on screening for *β*-lactamases, 293 isolates were positive for one of the three beta lactamases, ESBL, AmpC, or carbapnemases. Coproduction of ESBL and MBL was seen in 5 isolates, 35 isolates showed coproduction of ESBL and AmpC enzymes, and AmpC and MBL coproduction was exhibited in only in 5 isolates.

**Conclusions:**

Coexistence of ESBLs, AmpC producers, and carbapenemases has been described. Continuous monitoring and surveillance and proper infection control and prevention practices will limit the further spread of these superbugs within the hospital and beyond.

## 1. Introduction

Gram-negative bacteria (GNB) belonging to *Enterobacteriaceae* are known to cause serious infections, and treatment is often complicated because of the increasing bacterial resistance mediated by the presence of varying degrees of *β*-lactamase enzymes. It is not unusual to find a single isolate that expresses multiple *β*-lactamase enzymes, further complicating the treatment option [1, 2]. *Enterobacteriaceae* are associated with numerous infections such as UTI's, skin and soft tissue infection, and pneumonia among community-acquired infections [3, 4]. Aminoglycosides and third-generation cephalosporins are commonly used for treating infections caused by these organisms. These antibiotics are less effective as *β*-lactamase-producing isolates of *Enterobacteriaceae* are on the rise. Carbapenems are the current choice for treating the infection caused by extended spectrum beta lactamase (ESBL) producers; however, emergence of carbapenem-resistant isolates has also been noticed. Hence, the successful treatment outcome of these infections is seriously hampered by the presence of these enzymes [5, 6]. Carbapenem resistance in *Enterobacteriaceae* had been a negligible phenomenon before the start of this century. Back then, the rare occurrence of reduced susceptibility to carbapenems in *Enterobacteriaceae* was mostly attributed to a combination of production of ESBL or AmpC *β*-lactamase and deficiency of porins in the outer membrane [7, 8]. Induction of these *β*-lactamases may affect adversely the treatment of clinical conditions caused by such strains. Hence, the present study is designed to investigate the different *β*-lactamases and their coexistence among *Enterobacteriaceae* by using different detection methods.

## 2. Materials and Methods

### 2.1. Specimens, Inclusion Criteria, and Identification of *Enterobacteriaceae* Isolates

All types of nonrepetitive clinical specimens were received as part of standard patient care investigation from the out-patient department (OPD) and were processed for culture and antibiotic susceptibility testing. The patients already on antibiotics were excluded based on the history of antibiotics mentioned in the culture investigation form or with a prior history of hospitalization. All the organisms were isolated and identified using standard microbiological technique [9]. All specimens were cultured on MacConkey and blood agar and urine samples on CLED which then were incubated overnight at 37°C in the department of microbiology, using the standard microbiological technique [10]. On growth, these were subjected to various biochemical testing for identification. A Bact/Alert 3D system and VITEK 2 Compact were used where required. All isolates were subjected for antibiotic susceptibility testing using the Kirby Bauer Disk diffusion method as per CLSI 2013 guidelines [11].

### 2.2. Screening and Phenotypic Confirmation of ESBL, AmpC, and Carbapenemase Producers

All isolates were screened for ESBL production by using a disc diffusion test for of *Enterobacteriaceae*. In this test, a disc of ceftazidime (30 *µ*g), cefotaxime (30 *µ*g) alone, and a disc of ceftazidime and cefotaxime in combination with clavulanic acid (30/10 *µ*g) were used for each isolates. Both the discs were placed on a lawn culture of the test isolate on a Muller–Hinton agar plate and incubated overnight at 37°C. A ≥5 mm increase in zone diameter for either antimicrobial agent tested in combination with clavulanic acid versus its zone when tested alone was designated as ESBL positive [11]. For positive control *K. pneumoniae* ATCC 700603 and for Negative *E. coli* ATCC 25922 were used. A phenotypic confirmatory test for ESBL producers was done by a double disc diffusion test, for all the ESBL-producing isolates as per CLSI 2013 guidelines [11–13]. All isolates were subjected to screening for AmpC *β*-lactamase production using a 30 *µ*g cefoxitin disc (CX) by the disc diffusion method as per CLSI guidelines [11]. Confirmation of AmpC *β*–lactamases was done by the cefoxitin-cloxacillin double-disc synergy test (CC-DDS) [14, 15], and AmpC E-test was performed [16]. Screening for carbapenem-resistant GNB from the routine clinical samples was done by using 10 *μ*g imipenem discs (HiMedia). MHT, imipenem-EDTA disc method CDT [17], and E-test were performed on all imipenem-resistant isolates for phenotypic detection of carbapenemases.

### 2.3. Data Analysis

All the statistical analyses were carried out using Statistical Package for Social Sciences (SPSS) version 20.

## 3. Results

Four hundred and seventeen multidrug-resistant Gram-negative bacteria belonging to *Enterobacteriaceae* were reported from various clinical specimens during the period from May 2014 to June 2016. All such isolates were tested for antibiotic sensitivity [11].

Screening for *β*-lactamase enzymes like ESBLs, MBLs, and AmpC was performed using ceftazidime, imipenem, and cefoxitin respectively. Out of 417 isolates, 293 showed resistance to one of the three drugs. Of these 293 isolates, 283 isolates were resistant to ceftazidime, which were tested for ESBL production, 15 were resistant to imipenem, which were checked for carbapenemases production, and 114 isolates were resistant to cefoxitin, which were checked for AmpC production.

Results from Table 1 show that all 293 isolates were *β*-lactamase producers of which 21 organisms isolated from blood collected from patients with suspected enteric fever and screening for fever-related complaints were ESBL producers and 4 AmpC producers, 27 isolates from pus were ESBL, 2 MBL, and 3 AmpC producers, 35 isolates from sputum were ESBLs, 2 MBLs, and 6 AmpC producers, and 186 isolates from urine were ESBLs, 8 MBLs, and 40 AmpC producers.

Table 2 shows organism-wise distribution of *β*-lactamases (*n* = 293). All the isolates were screened for carbapenemase producers of which 15 isolates were imipenem-resistant organisms and 12 organisms were confirmed as MBL producers by Imipenem DDST and CDT tests. Remaining 3 of the imipenem-resistant isolates were MHT positive meaning they were not MBLs but some other carbapenemase producers. *E*. *coli* (40%) and *K*. *pneumoniae* (26.6%) were the 2 most common organisms exhibiting this phenomenon.

Table 2 shows the result of screening for AmpC producers using cefoxitin; 114 isolates showed resistance, and 53 were confirmed by DDST. Majority of the AmpC also belonged to *E*. *coli* (60.37%) and *K*. *pneumoniae* (26.41%).

283 (67.86%) isolates out of the 417 MDR were resistant to ceftazidime. *E*. *coli* and *K*. *pneumoniae* were the two most resistant ones.

All the Gram-negative organisms belonging to *Enterobacteriaceae* were subjected to screening tests using ceftazidime for ESBL production. 283 isolates were resistant to ceftazidime, and 269 (95.05%) of these were ESBL producers which were confirmed by (DDDT). *E*. *coli* 154 (54.41%) and *K*. *pneumoniae* 83 (29.32%) were the two most common isolates producing this enzyme.

Results after screening and confirmatory tests for CX (cefoxitin) Resistance and AmpC producers show 114 (27.33%) isolates out of the 417 MDR were resistant to Cefoxitin. *E*. *coli* and *K*. *pneumoniae* were the two most common organisms producing these enzymes.

Cefoxitin-resistant 114 isolates on confirmation by the cefoxitin-cloxacillin double disk synergy test (CC-DDS) and by imipenem E-strip test showed only 53 (46.49%) as AmpC producers. *E*. *coli* and *K*. *pneumoniae* were the two most common organisms with 32 (28.07%) and 14 (12.2%) isolates, respectively, whereas by an AmpC disk test, there were found out to be 48 (39.47%) cases.

Table 3 shows comparison between isolation of AmpC-producing organisms by the cefoxitin-cloxacillin disk diffusion test (CC-DDS), E-strip test, and AmpC disk test. AmpC producers by the CC-DDS and E-strip test were 53 while by AmpC disc test they were 48, which concluded that the CC-DDS and E-strip test are more sensitive tests. Confirmatory tests for MBL producer were carried out by the CDT, E-strip, and MHT test.Out of the 417 MDR, 15 (3.59%) isolates were resistant to imipenem. *E*. *coli* and *K*. *pneumoniae* were the two organisms producing these enzymes. Isolates which were resistant to imipenem were confirmed using the E-strip test, CDT, and MHT.

Table 4 shows results of screening and confirmatory tests for MBL detection in 15 isolates. Coproduction of ESBL and MBL was seen in 5 isolates out of which 2 isolates were of *E*. *coli*, 2 of *K*. *pneumoniae*, and 1 of *Proteus mirabilis.* When a chi-square test was performed to check if there was any statistical significance between organisms isolated and coproduction of ESBL and MBL, it was found that the *p*-value was more than 0.05, (*p*-value = 0.494) meaning coproduction of these enzymes was independent of the organisms isolated. Thirty five isolates showed coproduction of ESBL and AmpC enzymes, out of which 26 were *E*. *coli.* 4 isolates of *Klebsiella pneumonia,* 2 isolates each of *C*. *freundii* and *C*. *koseri* and 1 *Enterobacter* spp. showed coproduction.

### 3.1. Interpretation

It was found by the chi-square test for coproduction of ESBL and AmpC that it had a *p* value of less than 0.05 meaning it had a statistical significance, and coproduction of these two enzymes was organism dependent which in our study were *E*. *coli* and *K*. *pneumoniae* predominantly.

### 3.2. Coproduction of AmpC and MBL Was Exhibited by 5 Isolates of *E*. *coli*

#### 3.2.1. Interpretation

As *p* value is less than 0.05, it suggests that coproduction of MBL and AmpC enzymes is also organism dependent.

## 4. Discussion

This study was carried out with the intent to ascertain the isolation and identification of *β*-lactamase producers in GNB (*Enterobacteriaceae*) in samples from patients with community-acquired infections in a tertiary care center showing coexistence of *β*-lactamase enzymes in *Enterobacteriaceae*. In our study, we found 64.50% isolates to be pure ESBL producers and 12.70% isolates to be pure AmpC producers and 8.39% were ESBL **+** AmpC coproducers. Shoorashetty et al. reported 6% and 41% isolates to be pure AmpC and pure ESBL producers, respectively, with 27.5% isolates to be AmpC and ESBL coproducers [18]. Bakthavatchalu et al. reported 5.4% and 26.25% pure AmpC and pure ESBL producers, respectively, with 20.46% AmpC and ESBL coproducers [19]. Mohanty et al. reported 20.35% and 3.54% pure AmpC and pure ESBL producers, respectively, with 58.41% isolates to be AmpC ESBL coproducers [20]. Grover et al. reported 4.96 and 30.15% isolates to be pure AmpC and pure ESBL producers, respectively, with 9.92% isolates to be AmpC ESBL coproducers [14]. The coexistence of ESBL and MBL was reported in 5 isolates which contributed 1.7% of the total *β*-lactamases. This was slightly less than an other study by Loveena O et al. where the coproduction was seen in 8.79% isolates, whereas the AmpC and the MBL coproduction was shown by 1.7% isolates; in Loveena et al. study, the coproduction of AmpC and MBL was found to be 3.67%. A study which was done by Arora et al. reported AmpC and MBL coproduction in 46.6% isolates [21, 22]. The coexistence of ESBL and MBL was reported in 16% isolates, whereas the AmpC and the MBL coproduction was shown by 5% isolates, and the AmpC and the ESBL coproduction was shown in 24% isolates. A study which was performed by Arora et al. reported the AmpC and MBL coproduction in 46.6% isolates and the ESBL and AmpC coproduction in 3.3% isolates [22]. In our study, the ESBL and MBL coproduction was detected in 5 (1.7%) isolates and it was found in 2 isolates of *E. coli* and 2 of *K. pneumonia*, while the ESBL and the AmpC coproducers were 35 (8.39%) of the total MDR, and they were commonly isolated from *Escherichia coli* 26 (74.28%) of the total ESBL + AmpC followed by 4 isolates of *K*. *pneumonia*. The coproduction of AmpC and MBL was observed in 5 (1.7%) strains, and it was detected only in *E*. *coli* (100%). In an other study by Loveena et al., the ESBL and MBL coproduction was detected in 24 (8.79%) isolates and it was found to be maximum in *E. coli* (33.34%) and *K. pneumoniae* (16.67%), while the ESBL and the AmpC coproducers were 18 (6.59%), and they were commonly isolated from *E*. *coli* (50%) and *K*. *pneumoniae* (22.23%). The coproduction of AmpC and MBL was observed in 10 (3.67%) strains, and it was detected mostly in *E. coli* (60%) [21]. This study helps in knowing potentially resistant pathogens which can be encountered in community-based infections. Knowledge about these pathogens will help in early detection and treatment with proper antibiotics. These resistant organisms can be detected by various phenotypic detection methods as discussed above without going for genotypic methods. Coproduction of enzymes suggests that there is horizontal transfer of multiple resistance genes present in plasmids. This re-enforces the importance of continuous surveillance, especially of MDR *E*. *coli* and *K*. *pneumoniae* in the community, so that appropriate treatment can be administered. In the present study, it was seen that production of *β*-lactamases like ESBL and MBL is related to some organisms and statistically significant which was proved by chi-square tests. Hence, whenever such organisms are isolated, they should be screened for all *β*-lactamases like ESBL and MBL and dealt with proper antibiotics.

## 5. Conclusion

The high prevalence of *β*-lactamases and coexistence of ESBLs and carbapenemases were noted in *Enterobacteriaceae* isolates from community-acquired infections. Continuous monitoring of these *β*-lactamases and their coexistence will shed light in their dissemination and strategy to prevent and control the further spread of these superbugs.

## Figures and Tables

**Table 1:** Sample-wise distribution of *β*-lactamases (*n* = 293).

Samples	Frequency	Percentage (%)	ESBL	MBL	AmpC
Urine	198	67.6	186	8	40
Sputum	40	13.7	35	2	6
Pus	31	10.6	27	2	3
Blood	24	8.2	21	0	4
Total	293	100.0	269	12	53

**Table 2:** Organism-wise distribution of *β*-lactamases (*n* = 293).

Organisms	Frequency	Percent (%)	ESBL	MBL	AmpC
*E*. *coli*	161	54.9	154	6	32
*Klebsiella pneumonia*	86	29.4	74	4	14
*Citrobacter koseri*	20	6.8	19	0	3
*Citrobacter freundii*	15	5.1	14	0	3
*Proteus mirabilis*	7	2.4	6	1	0
*Proteus vulgaris*	2	0.7	1	0	0
*Enterobacter spp.*	1	0.3	1	0	1
*Klebsiella oxytoca*	1	0.3	0	1	0
Total	293	100.0	269	12	53

**Table 3:** Comparison between isolation of AmpC-producing organisms by the cefoxitin-cloxacillin disk diffusion test (CC-DDS), E-strip test, and AmpC disk test.

Organism	AmpC producer by AmpC disk test	AmpC E-strip test	AmpC producer by cefoxitin-cloxacillin disk diffusion test (CC-DDS)
*E*. *coli*	29	32	32
*Klebsiella pneumoniae*	12	14	14
*Citrobacter freundii*	3	3	3
*Citrobacter koseri*	3	3	3
*Enterobacter spp.*	1	1	1
Total	48	53	53

**Table 4:** Results of screening and confirmatory tests for MBL detection (*n* = 15).

Organism	I resistant (%)	CDT-positive isolates	E-strip test	MHT-positive isolates
*E. coli*	8 (53.3)	6 (40)	6 (40)	8 (53.3)
*Klebsiella pneumoniae*	5 (33.33)	4 (26.66)	4 (26.66)	5 (33.33)
*Klebsiella oxytoca*	1 (6.66)	1 (6.66)	1 (6.66)	1 (6.66)
*Proteus mirabilis*	1 (6.66)	1 (6.66)	1 (6.66)	1 (6.66)

## Data Availability

The data used to support the findings of this study are available from the corresponding author upon request.
